# Insights into the complex regulation of *rpoS* in *Borrelia burgdorferi*

**DOI:** 10.1111/j.1365-2958.2007.05813.x

**Published:** 2007-07-01

**Authors:** Mary N Burtnick, Jennifer S Downey, Paul J Brett, Julie A Boylan, Jonathan G Frye, Timothy R Hoover, Frank C Gherardini

**Affiliations:** 1 Laboratory of Zoonotic Pathogens, Rocky Mountain Laboratories, National Institute of Allergy and Infectious Diseases, National Institutes of Health Hamilton, MT, USA.; 2 Division of Diagnostic Science, Norris School of Dentistry, University of Southern California Los Angeles, CA, USA.; 3 USDA-ARS-SAA-RRC, Bacterial Epidemiology and Antimicrobial Resistance Research Unit Athens, GA, USA.; 4 Department of Microbiology, University of Georgia Athens, GA, USA.

## Abstract

Co-ordinated regulation of gene expression is required for the transmission and survival of *Borrelia burgdorferi* in different hosts. The sigma factor RpoS (σ^S^), as regulated by RpoN (σ^54^), has been shown to regulate key virulence factors (e.g. OspC) required for these processes. As important, multiple signals (e.g. temperature, pH, cell density, oxygen) have been shown to increase the expression of σ^S^-dependent genes; however, little is known about the signal transduction mechanisms that modulate the expression of *rpoS*. In this report we show that: (i) *rpoS* has a σ^54^-dependent promoter that requires Rrp2 to activate transcription; (ii) Rrp2Δ123, a constitutively active form of Rrp2, activated σ^54^-dependent transcription of *rpoS/P-lacZ* reporter constructs in *Escherichia coli*; (iii) quantitative reverse transcription polymerase chain reaction (QRT-PCR) experiments with reporter *cat* constructs in *B. burgdorferi* indicated that Rrp2 activated transcription of *rpoS* in an enhancer-independent fashion; and finally, (iv) *rpoN* is required for cell density- and temperature-dependent expression of *rpoS* in *B. burgdorferi*, but histidine kinase Hk2, encoded by the gene immediately upstream of *rrp2*, is not essential. Based on these findings, a model for regulation of *rpoS* has been proposed which provides mechanisms for multiple signalling pathways to modulate the expression of the σ^S^ regulon in *B. burgdorferi*.

## Introduction

*Borrelia burgdorferi,* the Lyme disease agent, is maintained in nature in two very distinct environments, *Ixodes* ticks and mammalian hosts (Burgdorfer *et al*., 1982; Steere *et al*., 1983). As *B. burgdorferi* moves from one host to another, its ability to adapt and survive is attributed to its capacity to sense changes in temperature, pH, cell density, oxygen and/or exposure to host factors and alter gene expression accordingly (Schwan *et al*., 1995; Stevenson *et al*., 1995; Indest *et al*., 1997; Ramamoorthy and Philipp, 1998; Carroll *et al*., 1999; Indest and Philipp, 2000; Schwan and Piesman, 2000; Ojaimi *et al*., 2003; Seshu *et al*., 2004). Previous reports have demonstrated that central to the regulation of these adaptive responses are σ^S^ (encoded by *rpoS*) and σ^54^ (encoded by *rpoN*, also known as *ntrA*). In addition, σ^54^-dependent expression of σ^S^ is responsible for the expression of key virulence factors [e.g. outer surface protein C (OspC), OspA and decorin binding protein A (DbpA)] required for infectivity and transmission during the infective cycle (Elias *et al*., 2000; Yang *et al*., 2000; Hübner *et al*., 2001; Caimano *et al*., 2004; Fisher *et al*., 2005).

σ^54^-dependent transcription is a tightly regulated process requiring an activator protein that stimulates the isomerization of the closed σ^54^-RNAP (RNA polymerase) holoenzyme/promoter complex to the open complex in a reaction that requires ATP hydrolysis by the activator (Sasse-Dwight and Gralla, 1988; Popham *et al*., 1989). In general, σ^54^-dependent activators are modular in structure consisting of an N-terminal regulatory domain, a central ATPase domain and a C-terminal DNA binding domain (North *et al*., 1996; Ogura and Wilkinson, 2001; Xu and Hoover, 2001). These activators bind enhancer-like sequences upstream of their target promoters and then contact the closed complexes through DNA looping (Su *et al*., 1990). In *B. burgdorferi*, the two-component response regulator Rrp2 (BB0763) is a σ^54^-dependent activator that controls *rpoS* expression (Yang *et al*., 2003a). Attempts to disrupt *rrp2* have been unsuccessful so far, but a single amino-acid change within the ATPase domain of Rrp2 eliminates σ^S^-dependent lipoprotein expression by disrupting the cascade involved in the σ^54^-dependent expression of *rpoS* (Yang *et al*., 2003a). Because both *B. burgdorferi rpoN* and *rpoS* mutants are viable (Elias *et al*., 2000; Hübner *et al*., 2001; Fisher *et al*., 2005), the inability to generate Rrp2-deficient mutants suggests that Rrp2 has an essential function in *B. burgdorferi* that is separate from its role in activating transcription of *rpoS* (Yang *et al*., 2003a).

The gene immediately upstream of *rrp2*, designated *hk2* (BB0764), is predicted to encode a histidine kinase through which sensory information is channelled to activate Rrp2 (Yang *et al*., 2003a). Hk2 is thought to donate phosphate to Rrp2 to stimulate its activity; however, the specific signals that trigger the autophosphorylation of Hk2 and influence its interactions with Rrp2 have not been identified. Another intriguing aspect of the σ^54^–σ^S^ regulatory pathway is that multiple signals (e.g. temperature, pH and cell density) seem to simulate the expression of the σ^S^-dependent genes, suggesting that additional systems influence the transcriptional control of *rpoS*.

In this article, we report the identification of the *rpoS* transcriptional start site downstream of a σ^54^-dependent promoter and reveal characteristics of Rrp2 that potentially explain how this protein plays a more complex role in transcriptional regulation of *rpoS.* In addition, we constructed an *hk2* mutant and assessed the involvement of this putative histidine kinase with regard to *rpoS* and *ospC* expression. Based upon our findings in the present study, we put forward a model which may explain how multiple signalling pathways control the expression of σ^S^ and its regulon in *B. burgdorferi.*

## Results

### Confirmation of the *σ*^54^-dependent *rpoS* promoter

**Fig. 1:**
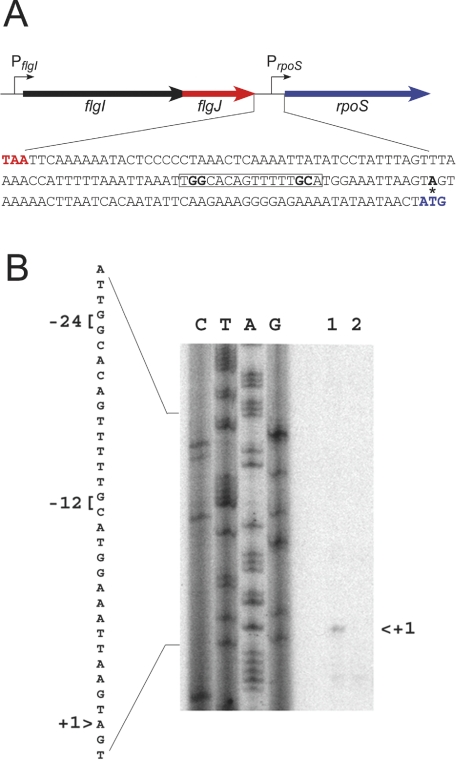
Primer extension analysis of *rpoS* transcripts A. Schematic of the *B. burgdorferi rpoS* region indicating *flgI* (black arrow), *flgJ* (red arrow) and *rpoS* (blue arrow); the approximate locations of putative promoters (small bent black arrows) are indicated. The gene names are as annotated in the *B. burgdorferi* B31 genome sequence (TIGR and/or ERGO, Integrated Genomics). The DNA sequence in the region between *flgJ* and *rpoS* is shown. The stop codon (TAA) for *flgJ* is indicated in red, and the start codon (ATG) for *rpoS* is indicated in blue. The σ^54^-dependent promoter for *rpoS* is highlighted with a box around it, and the conserved −24/−12 GG/GC doublets are shown in bold. The transcriptional start site for this promoter is indicated in bold with an asterisk below it. B. Primer extension reactions were performed with RNA extracted from *B. burgdorferi* strains B31-A (1) and B31-ARpoN (2) at 2 × 10^8^ cells ml^−1^. The sequencing ladder was generated with the rpoS-4 primer. The −24/−12 doublets are indicated, and the +1 site is indicated by an arrowhead (<) denoting the 5′ end of the transcript.

Several studies have shown that *B. burgdorferi rpoS* expression is regulated by σ^54^ (Hübner *et al*., 2001; Yang *et al*., 2003a,b; Fisher *et al*., 2005). In *B. burgdorferi* B31, a potential σ^54^-dependent promoter is located 62 bp upstream of the putative *rpoS* start codon (Fig. 1A) (Buck *et al*., 2000; Hübner *et al*., 2001). Primer extension reactions using RNA isolated from *B. burgdorferi* strain B31-A (high-passage) cells grown to high density (2 × 10^8^ cells ml^−1^) revealed an extension product that terminated 14 bp downstream of the GC doublet of the putative σ^54^-dependent promoter sequence (Fig. 1B). *B. burgdorferi* strain B31-ARpoN is a derivative of B31-A in which *rpoN* is disrupted with a kanamycin-resistance cassette. RNA isolated from B31-ARpoN cells grown to high density (2 × 10^8^ cells ml^−1^) failed to yield an extension product from the σ^54^-dependent promoter (Fig. 1B). Taken together, these observations strongly suggest that the sequence containing a predicted −24/−12 region upstream of *rpoS* is a σ^54^-dependent promoter. These results are consistent with those recently reported by both Smith *et al*. (2007) and Lybecker and Samuels (2007), which employed 5′-RACE analysis to identify the identical transcriptional start site in the infectious clone *B. burgdorferi* BbAH130 and low-passage strain 297, respectively.

**Fig. 2:**
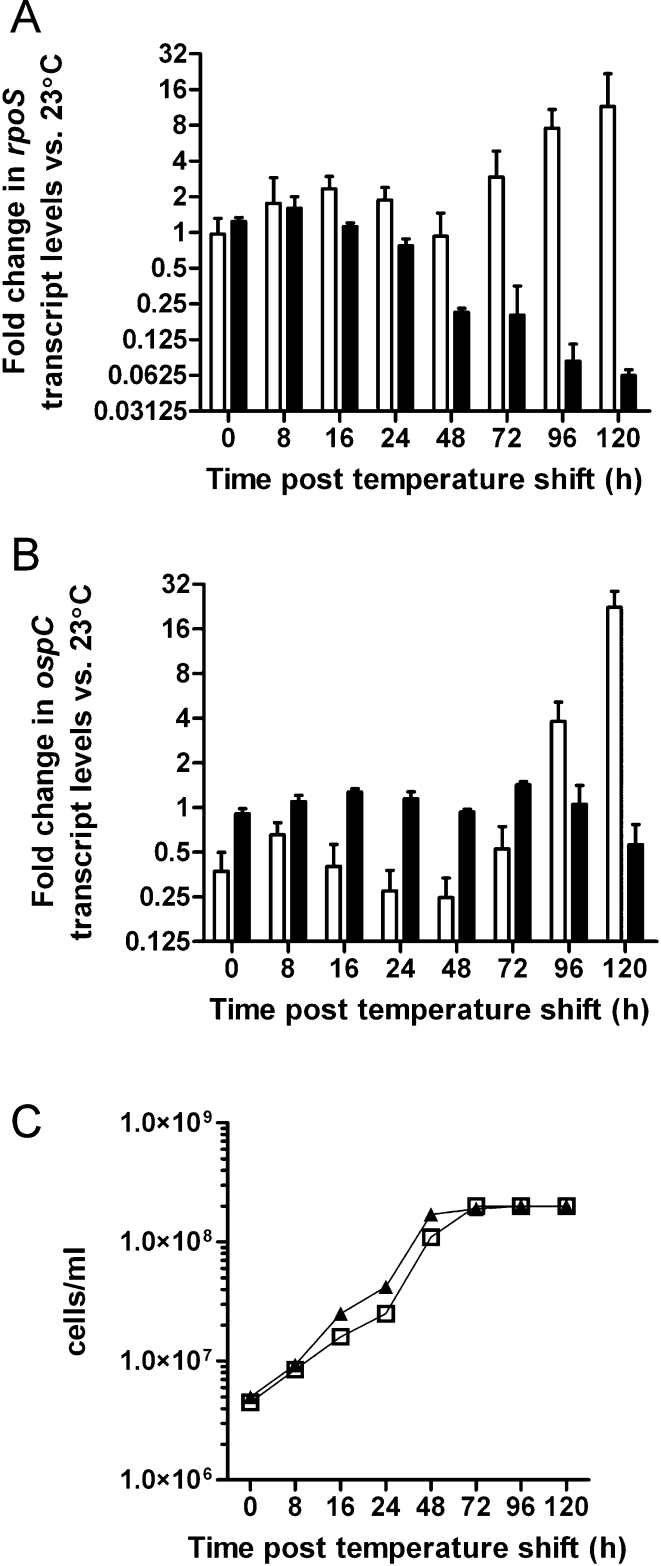
Quantitative RT-PCR analysis of *rpoS* and *ospC* transcripts following an increase in growth temperature from 23°C to 34°C. RNA was extracted from *B. burgdorferi* strains B31-A3 (low-passage, white bars) and B31-A (high-passage, black bars) grown at 23°C, and at various time points following a temperature shift to 34°C. Levels of transcripts were measured with specific primer/probe sets using Taqman, and values have been normalized to the internal control, *flaB.* Data presented represents averages of three assays performed in quadruplicate. Fold changes are expressed relative to spirochetes grown at 23°C. Error bars represent standard deviation. A. QRT-PCR analysis of *rpoS* following a temperature shift. B. QRT-PCR analysis of *ospC* following a temperature shift. C. Growth curves of B31-A3 (white squares) and B31-A (black triangles) following a temperature shift from 23 to 34°C.

Although the σ^54^-dependent transcriptional start site for *rpoS* maps to the same location in both high- and low-passage *B. burgdorferi* strains, previous studies have demonstrated that the RpoN/RpoS pathway may be defective in high-passage isolates (Masuzawa *et al*., 1994; Elias *et al*., 2000; Schwan and Piesman, 2000; Lybecker and Samuels, 2007). To determine whether this pathway is functioning properly in high-passage *B. burgdorferi* strain B31-A, we conducted a side-by-side comparison of *rpoS* and *ospC* transcript levels in B31-A (high-passage) and B31-A3 (low-passage) at various time points following a temperature shift from 23°C to 34°C. Results of quantitative reverse transcription polymerase chain reaction (QRT-PCR) experiments demonstrated an increase in *rpoS* and *ospC* expression following an increase in growth temperature, indicating that the RpoN/RpoS pathway functions as expected in strain B31-A3 (Fig. 2). However, in B31-A, *rpoS* and *ospC* transcript levels initially remained unchanged following a temperature shift but decreased as cell density increased, indicating that temperature regulation was defective in this high-passage strain (Fig. 2). Based upon these results, all further experiments in this study were conducted with low-passage *B. burgdorferi* strain B31-A3 and its derivatives.

### Purified Rrp2 and Rrp2Δ123 fail to bind DNA sequences immediately upstream of *B. burgdorferi rpoS*

Purification of recombinant Rrp2 from *Escherichia coli* for *in vitro* studies was complicated by poor expression and insolubility. Additionally, initial attempts to purify recombinant Rrp2 resulted in proteins with incorrect amino-acid sequences as determined by N-terminal sequencing (data not shown). Assessment of the *rrp2* codon usage using GCUA Graphical Codon Usage Analyzer (http://gcua.schoedl.de/) revealed that approximately 38% of the codons in this open reading frame (ORF) were rare in *E. coli,* which likely accounted for the poor expression of Rrp2 in *E. coli* (Boylan *et al*., 2006). As an alternative, a codon-optimized version of *rrp2*, designated *rrp2op*, was synthesized. Because efficient binding of σ^54^-dependent activators may require phosphorylation of the N-terminal receiver domain (Lee *et al*., 1994; 2003), a truncated version of *rrp2op* was polymerase chain reaction (PCR) amplified by deleting the first 369 nucleotides (corresponding to 123 codons). Both the full-length and truncated genes were cloned into pBAD-TOPO and overexpressed in *E. coli*. The expression of the truncated gene was expected to result in a constitutively active form of Rrp2 (designated Rrp2Δ123) consisting of the central ATPase and DNA binding domains. Rrp2 and Rrp2Δ123were purified to homogeneity from the soluble fraction as assessed by SDS-PAGE using heparin–sepharose affinity chromatography (data not shown). The majority of Rrp2 and Rrp2Δ123 eluted from the heparin column in the 200–300 mM KCl fractions (data not shown). Both forms of Rrp2 were used in DNA mobility-shift assays.

**Fig. 3:**
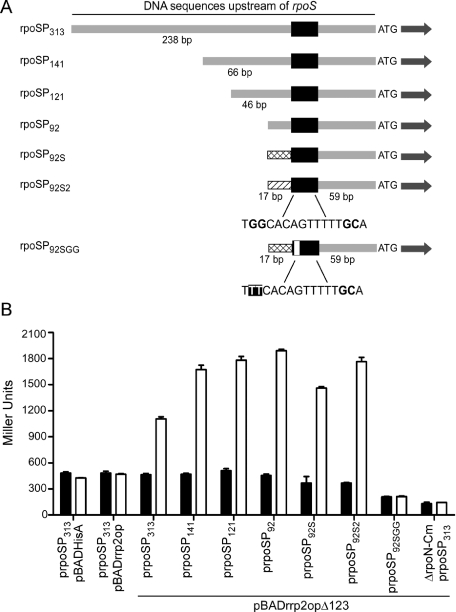
A. Schematic representation of the promoter regions upstream of *B. burgdorferi rpoS* used for mobility-shift DNA binding assays and transcriptional fusion analyses. The labels on the left indicate the names of the *rpoS* promoter regions cloned into the promoter-less *lacZ* or *cat* reporter plasmids. The start of *rpoS* is indicated by an ATG codon. The grey lines to the left of the ATG represent the DNA upstream of *rpoS*. The black boxes indicate the location of the σ^54^-dependent promoter; the exact sequence of this promoter is shown below with the characteristic −12/−24 GC/GG bases shown in bold. The number of base pairs shown on either side of the black boxes specifies the length of the DNA fragment on either side of the σ^54^-dependent promoter (e.g. 238 bp upstream of the −24 or 59 bp downstream of the −12). The hatched lines shown on rpoSP_92S_ and rpoSP_92S2_ indicate the DNA sequences that were randomly scrambled; exact sequences for these scrambled regions are shown in Table 2. The rpoSP_92SGG_ fragment is identical to the rpoSP_92S_; however, it harbours a mutation in the σ^54^-dependent promoter at the −24 position (GG to TT) as indicated in white. B. Transcription from the *rpoS* promoter region is activated by Rrp2Δ123. β-Galactosidase (LacZ) activity was measured in *E. coli* Top 10 cells harbouring reporter constructs and expression vectors. *lacZ*-fusion plasmids harbouring varying-length DNA fragments encompassing the *B. burgdorferi rpoS* promoter region are indicated, along with either pBADHisA (vector control), pBADrrp2op (expresses full-length Rrp2), or pBADrrp2opΔ123 (expresses N-terminally truncated Rrp2, designated Rrp2Δ123). *E. coli*ΔrpoN-Cm, an *rpoN* mutant, is indicated. Assays were performed after 6 h growth in LB media without (black bars) or with 0.002% l-arabinose (white bars) to induce protein expression. Error bars represent standard deviation.

To identify a potential enhancer Rrp2 binding sequence upstream of *rpoS*, two DNA fragments, rpoSP_313_ (313 bp) and rpoSP_141_ (141 bp) (Fig. 3A), were amplified by PCR for use in gel mobility-shift assays. Each fragment consisted of the σ^54^-dependent *rpoS* promoter along with 59 bp of downstream DNA and either 238 or 66 bp of upstream DNA respectively. Because most σ^54^-dependent activators bind within 200 bp of their target promoters, the rpoSP_313_ fragment was predicted to contain enough upstream DNA sequence to include a potential enhancer. Despite numerous attempts, under a variety of binding conditions, no binding of Rrp2 or Rrp2Δ123 to either rpoSP_313_ or rpoSP_141_ was observed. These observations suggested that there is no high-affinity Rrp2 binding site within the 238 bp upstream of the *rpoS* promoter. Alternatively, it is possible that the correct conditions for binding of Rrp2 or Rrp2Δ123 were not satisfied in the *in vitro* assay.

**Table 2:** Polymerase chain reaction (PCR), PE, RT-PCR and QRT-PCR primers and probes used in this study.

Primer name	Primer sequence (5′→ 3′)^a^–^d^
PCR and PE primers	
rpoS-4	TATGTTTAAATCAGCTTAATTTATTTC
up-PE	TAGATTTTGTGAATTAATTTTGGTTTCC
Rrp2op-F	ATGAGCAAAATTCTGGTGGCGG
Rrp2op-R	TTAATCAATATTATATTCGATAA
Rrp2opΔ123-F	GATAATGAAAATGCGAATCTGGAAAATATTCTG
rpoSP313-FB	AAGCGGATCCTTGAAAGCATGAAAAAA
rpoSP-RXhoI	CCGCTCGAGTATTATATTTTCTCCCCTT
rpoSP141-FB	AAGCGGATCCCAAAAAATACTCCCCCT
rpoSP121-FB	AAGCGGATCCCTCAAAATTATATCCTATTTAG
rpoSP92-FB	AAGCGGATCCCCATTTTTAAATTAAATTGGCAC
rpoSP92S-FB	AAGCGGATCC*CACATTATTATAATATA*TGGCACAGTTTTTGCATG
rpoSP92S2-FB	AAGCGGATCC*ACCTATAAATTATATTA*TGGCACAGTTTTTGCATG
rpoSP92SGG-FB	AAGCGGATCC*CACATTATTATAATATA*TTTCACAGTTTTTGCATG
rpoSP92-RXb	GTACTCTAGATATTATATTTTCTCCCCTT
rpoSP92S-FH	GATCAAGCTT*CACATTATTATAATATA*TGGCACAGTTTTTGCATG
rpoSP313-FH	GATCAAGCTTCAAAAAATACTCCCCCT
bosRPO-FB	AAGCGGATCCAATTGAAAAAATAAATTCTAAGAAATGG
bosRPO-RX	CCGCTCGAGATGATTATACCTTTTTTGTTTAAATTAAAG
EcrpoN-F	ATGAAGCAAGGTTTGCAACTCAGGCTTAGCCAACAACTGGGTGTAGGCTGGAGCTGCTTC
EcrpoN-R	TCAAACGAGTTGTTTACGCTGGTTTGACGGCGGAATGGATCATATGAATATCCTCCTTA
c1	TTATACGCAAGGCGACAAGG
yhbG-430	GCCGTGTAGAAATTGCCCGC
hk2SphF	GTGGCATGCAGTGTTACAACCAATTAACC
hk2SphR	GTGGCATGCAATCTCTGATGTTACATTGC
hk2-BF	GTGGGATCCATCGCCCCTATAATCAAAAT
hk2-BR	GTGGGATCCAGCTTCTTCTTCGTCACTA
kan5′	GCAATGTAACATCAGAGATT
kan3′	GGTTAATTGGTTGTAACACT
RT-PCR, QRT-PCR	
primers and probes	
rrp2-RTF	GGCTGCAACAAACAAAAACATT
rrp2-RTR	TTTCATTGCATCATTAGAAAGAGTT
flaB-586F	AATCTTTTCTCTGGTGAGGGAGCT
flaB-657R	TCCTTCCTGTTGAACACCCTCT
flaB-611T	**FAM**^c^-AAACTGCTCAGGCTGCACCGGTTC-**TAMRA**^d^
cat-284F	ACAAGGGTGAACACTATCCCATATC
cat-256R	GAATGCTCATCCGGAATTACG
cat-310T	**FAM**^c^-CCAGCTCACCGTCTTTCATTGCCA-**TAMRA**^d^
rpoS-215F	AAGAAGGCAACTTGGGATTAATAAGA
rpoS-308R	TGCTTAATCCAAAATGATGCATAAG
rpoS-242T	**FAM**^c^-CTGCTGAAAAATATGACCCGAATAAAAATACCAAATT-**TAMRA**^d^
ospCRT-F1	ACGGATTCTAATGCGGTTTTACTT
ospCRT-R1	CAATAGCTTTAGCAGCAATTTCATCT
ospCRT-P	**FAM**^c^-ATGACAGCAACGCTTCAACCTCTTTCACAG-**TAMRA**^d^

a Linker sequences are underlined.

b. Scrambled sequences are italicized.

c **FAM** = 6-carboxyflourescein.

d **TAMRA** = 5-carboxytetramethylrhodamin

### Rrp2Δ123 functions with *E. coli* σ^54^-holoenzyme to activate transcription from the *rpoS* promoter

Due to our inability to generate an *rrp2* mutant in *B. burgdorferi*, we decided to use *E. coli* as a surrogate system to assess Rrp2-dependent activation of transcription from the *B. burgdorferi rpoS* promoter region. Rrp2 activity was examined in *E. coli* using a reporter construct in which the *B. burgdorferi rpoS*σ^54^-dependent promoter, along with 59 bp of downstream DNA sequence and 238 bp of upstream DNA sequence (rpoSP_313_ fragment), was introduced upstream of a promoter-less *lacZ* in plasmid pPBMB101. This reporter plasmid, designated prpoSP_313_, was introduced into an *E. coli* Top 10 strain that expressed full-length Rrp2 or Rrp2Δ123. Because the levels of σ^54^ in *E. coli* remain relatively constant throughout growth phases (Jishage *et al*., 1996), we chose to assess promoter activity at 6 h following the addition of arabinose to allow for sufficient Rrp2 expression as confirmed by immunoblot analysis (data not shown). Full-length Rrp2 did not activate transcription from the *rpoSP-lacZ* reporter gene above background levels (i.e. in the absence of any Rrp2 protein) even when expression of the activator was induced with arabinose (Fig. 3B). This observation was expected because Rrp2 presumably must be phosphorylated to be active.

Induction of Rrp2Δ123 expression with arabinose, in contrast, stimulated expression of the *rpoSP-lacZ* reporter construct, prpoSP_313_ (Fig. 3B). A set of *rpoSP-lacZ* reporter genes with varying lengths of DNA (17–238 bp) upstream of the *rpoS* promoter were constructed and examined to determine whether RrpΔ123-mediated transcriptional activation from the *rpoS* promoter in *E. coli* required an upstream enhancer (Fig. 3A). Induction of Rrp2Δ123 expression with arabinose led to approximately a 3- to 4-fold increase in LacZ activity for all *rpoSP–lacZ* fusions harbouring an intact σ^54^-dependent promoter compared with no inducer (Fig. 3B). Similarly, two reporter constructs, prpoS_92S_ and prpoS_92S2_, that included 17 bp of randomly scrambled DNA sequence immediately of upstream of the σ^54^-dependent promoter (Fig. 3A) also demonstrated approximately a fourfold increase in LacZ activity in the presence of Rrp2Δ123 (Fig. 3B). In contrast, the prpoS_92SGG_ plasmid, in which the σ^54^-dependent promoter was mutated at the −24 position (GG to TT), showed no increase in LacZ activity in the presence of inducer (Fig. 3B), indicating that the promoter must be intact for transcriptional activation. Similarly, the levels of LacZ activity from the prpoSP_313_ reporter plasmid in an *E. coli rpoN* mutant did not increase with the addition of arabinose, indicating that the Rrp2Δ123-mediated transcriptional activation was dependent on σ^54^ (Fig. 3B). A recent report by Smith *et al*. (2007) demonstrating that *E. coli* RpoN is capable of binding upstream of the *B. burgdorferi rpoS* gene supported our data indicating that Rrp2Δ123-mediated transcriptional activation in our reporter system was dependent on *E. coli*σ^54^. Expression from the *bosR*PO-*lacZ*, was not affected by Rrp2Δ123 (data not shown) as expected, because transcription of *bosR* is not dependent on σ^54^ (Fisher *et al*., 2005). Taken together, these data suggest that the 238 bp of DNA sequence upstream of the σ^54^-dependent *rpoS* promoter does not harbour an enhancer binding sequence recognized by Rrp2Δ123 for transcriptional activation in *E. coli*, which is consistent with the results of the mobility-shift DNA binding assays.

### Rrp2 does not require upstream DNA sequences for transcriptional activation from the *rpoS* promoter in *B. burgdorferi*

**Fig. 4:**
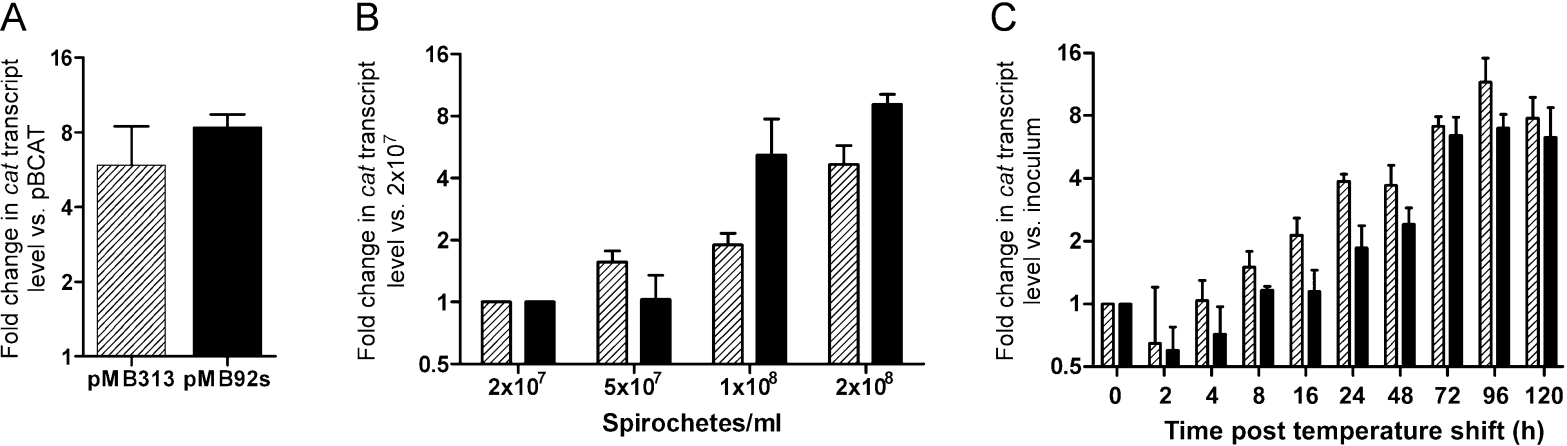
Transcript levels of *cat* in *B. burgdorferi* B31-A3 as measured by QRT-PCR. All values have been normalized to the internal control, *flaB*. Error bars represent standard deviation A. *cat* transcripts levels were measured in *B. burgdorferi* A3 harbouring *cat* reporter plasmids pMB313 (rpoSP_313_ fragment), pMB92S (rposP_92S_ fragment) and pBCAT (vector control) at a cell density of 2 × 10^8^ cells ml^−1^. Fold changes are relative to the vector control strain. B. *cat* transcripts levels were measured in *B. burgdorferi* B31-A3 harbouring *cat* reporter plasmids pMB313 (hatched bars) and pMB92S (black bars) at varying cell densities. Fold changes are relative to the 2 × 10^7^ spirochetes ml^−1^ culture. C. *cat* transcripts levels were measured in *B. burgdorferi* B31-A3 harbouring *cat* reporter plasmids pMB313 (hatched bars) and pMB92S (black bars) following an increase in growth temperature from 23°C to 34°C. Fold changes are relative to the inoculums used at *t* = 0 h.

Previous studies have demonstrated that when produced in excess, σ^54^-activator proteins (e.g. NtrC or N-terminally truncated DctD) can lead to activation of transcription from solution (Lee *et al*., 1994; North and Kustu, 1997). Therefore, to confirm that overexpression of RrpΔ123 was not responsible for the observed activation of the *rpoS* promoter constructs in *E. coli*, two reporter plasmids were constructed fusing the *rpoS*σ^54^-dependent promoter and either 238 bp (pMB313) or 17 bp (pMB92S) of upstream DNA to a promoter-less *cat* gene in plasmid pBCAT, and were transformed into *B. burgdorferi*. This allowed for assessment of Rrp2-mediated transcriptional activation of *rpoS* in the presence of wild-type levels of Rrp2. Levels of *cat* expression from the two reporter constructs in *B. burgdorferi* B31-A3 (2 × 10^8^ cells ml^−1^) were monitored by QRT-PCR and compared with that of pBCAT. Results indicated a 7-fold increase in *cat* expression in the strain harbouring pMB313 and a 9-fold increase in *cat* expression in the strain harbouring pMB92S compared with the same strain harbouring the pBCAT vector (Fig. 4A). Further experiments monitored the levels of *cat* expression from the two reporter plasmids as *B. burgdorferi* cell density increased or following a temperature shift from 23°C to 34°C. QRT-PCR results demonstrated that *cat* transcript levels increased 6- to 8-fold as spirochete density increased (Fig. 4B). Similarly, temperature-shift experiments showed that *cat* transcript levels increased as much as 8-fold following an increase in growth temperature (Fig. 4C) Taken together, these data indicated that, as observed with Rrp2Δ123 in *E. coli*, the 238 bp of DNA sequence immediately upstream of the *rpoS*σ^54^-dependent promoter is not required for activation of *rpoS* transcription by Rrp2 in *B. burgdorferi*.

### Construction of a *B. burgdorferi hk2* mutant

**Fig. 5:**
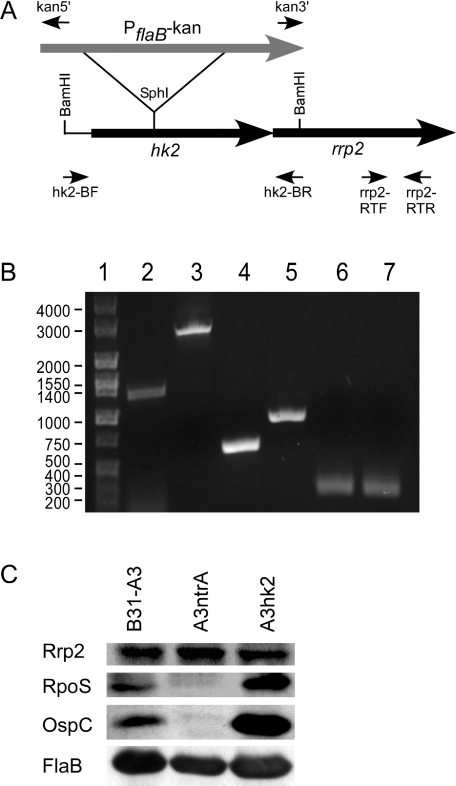
Construction of a *B. burgdorferi hk2* mutant A. Schematic representation for inactivation of *hk2* in B31-A3. *hk2* and *rrp2* are represented by black arrows as labelled. A DNA fragment harbouring *hk2* was PCR amplified using hk2-BF and hk2-BR primers and insertionally disrupted at a unique SphI site with a kanamycin cassette (grey arrow) as described in the *Experimental procedures* section. Primers are denoted by short black arrows. B. Agarose gel patterns of PCR products for B31-A3 (lane 2) and A3*hk2* (lane 3) using the hk2-BF and hk2-BR primer pair. Disruption of *hk2* by the kanamycin cassette resulted in an increased size PCR product (compare lanes 2 and 3). PCR products for the hk2-BF and kan5′ primer pair (lane 4), and the hk2-BR and kan3′ primer pair (lane 5), confirmed the orientation of the kanamycin cassette with respect to *hk2* and *rrp2* as diagrammed in panel A. RT-PCR analysis with the rrp2-RTF and rrp2-RTR primer pair confirmed the presence of *rrp2* transcript in both B31-A3 (lane 6) and A3*hk2* (lane 7). Lane 1 contains DNA markers with the sizes indicated to the left. C. Immunoblot analysis of B31-A3, A3*ntrA* and A3*hk2* grown to high cell density (2 × 10^8^ cells ml^−1^ + 24 h). Whole-cell lysates of *B. burgdorferi* strains equivalent to ∼10^8^ cells were separated on a 12% Tris-glycine gel, immobilized on a nitrocellulose membrane and probed with antiserum specific for the antigens indicated on the left. FlaB serves as a loading control to demonstrate equivalent protein amounts between samples.

The gene immediately upstream of *rrp2*, *hk2*, encodes the putative cognate histidine kinase for Rrp2. Consistent with this prediction, purified Hk2 can autophosphorylate and subsequently donate phosphate to Rrp2, although this phospho-transfer is inefficient *in vitro* (unpublished data). To determine the influence of Hk2 on *rpoS* expression, an *hk2* mutant in *B. burgdorferi* low-passage strain B31-A3 (designated as strain A3*hk2*) was generated by disrupting the gene with a kanamycin-resistance cassette (Fig. 5A). The disruption of *hk2* in A3h*k2* was confirmed by PCR (Fig. 5B). The kanamycin-resistance cassette was inserted into *hk2* so that its promoter (P_*flaB*_) would allow for *rrp2* expression, and the orientation of this insertion was confirmed by PCR (Fig. 5B). Subsequently, reverse transcription polymerase chain reaction (RT-PCR) using the rrp2-RTF and rrp2-RTR primer pair confirmed the presence of *rrp2* transcript in both B31-A3 and A3*hk2* (Fig. 5B). The levels of Rrp2 in B31-A3 and A3*hk2* were equivalent as assessed by immunoblot analysis (Fig. 5C), confirming that insertion of the kanamycin-resistance cassette into *hk2* was not polar on *rrp2*. *B. burgdorferi* A3*ntrA*, an *rpoN* mutant, was also shown to express similar levels of Rrp2. The plasmid content of A3*hk2* was assessed and, in comparison with the parent strain, was observed to be missing lp21 and lp36.

Because Hk2 is predicted to phosphorylate Rrp2, which subsequently governs σ^54^-dependent RpoS and OspC production, the expression of these proteins was assessed in A3*hk2*. Spirochetes were grown to high density (2 × 10^8^ cells ml^−1^), and immunoblotting was used to determine levels of RpoS and OspC compared with the parent strain B31-A3 and A3*ntrA* (negative control). Results showed that A3*hk2* appeared to express increased levels of both RpoS and OspC compared with B31-A3, while neither protein was detected in A3*ntrA* (Fig. 5C). These data indicated that expression of these two proteins was σ^54^-dependent, but not Hk2-dependent.

### *rpoS* mRNA and RpoS levels in *B. burgdorferi* cultures indicate that *rpoN* is required, but *hk2* is not essential, for maximal expression of *rpoS* at high cell densities

Previous studies have demonstrated that *B. burgdorferi* cell density influences the expression of RpoS-controlled genes, including *ospC* (Indest *et al*., 1997; Ramamoorthy and Philipp, 1998; Caimano *et al*., 2004). In order to examine the role of an *hk2* mutation on the transcription of *rpoS* and *ospC*, levels of both *rpoS* and *ospC* transcripts were monitored using QRT-PCR in *B. burgdorferi* strains B31-A3, A3*ntrA* and A3*hk2* as cultures of these strains transitioned from lower density (4 × 10^6^ cells ml^−1^) to higher density (2 × 10^8^ cells ml^−1^). A3*ntrA* was used as a control to confirm that σ^54^ was required for expression of *rpoS* when cells reach high density (2 × 10^8^ cells ml^−1^) in low-passage *B. burgdorferi* (Fisher *et al*., 2005). Additionally, RpoS and OspC levels were determined using immunoblot analysis of whole-cell lysates corresponding to each time point at which transcript was measured.

**Fig. 6:**
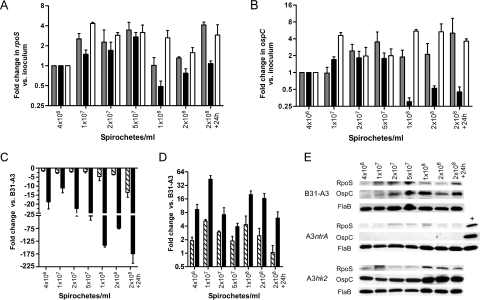
Quantitative RT-PCR analysis of *rpoS* and *ospC* transcripts and immunoblot analysis of RpoS and OspC as cell density increases RNA was extracted from *B. burgdorferi* strains B31-A3 (grey bars), A3*ntrA* (black bars) and A3*hk2* (white bars) as spirochete density increased and transcripts were quantified using specific primers and probes with the Taqman system. Values have been normalized to the internal control, *flaB.* Data presented represents averages of three assays performed in quadruplicate. Error bars represent standard deviation. A. QRT-PCR analysis of *rpoS* as cell density increased. Fold changes are expressed relative to the initial inoculum. B. QRT-PCR analysis of *ospC* as cell density increased. Fold changes are expressed relative to the initial inoculum. C. QRT-PCR analysis of *rpoS* (hatched bars) and *ospC* (black bars) transcripts in A3*ntrA* relative to B31-A3. Fold changes are expressed compared with B31-A3 at corresponding cell densities. D. QRT-PCR analysis of *rpoS* (hatched bars) and *ospC* (black bars) transcripts in A3*hk2* relative to B31-A3. Fold changes are expressed compared to the B31-A3 at corresponding cell densities. E. Immunoblot analysis of RpoS and OspC levels in *B. burgdorferi* strains B31-A3, A3*ntrA* and A3*hk2* as cell density increased. Whole-cell lysates of *B. burgdorferi* strains equivalent to approximately 8 × 10^7^−1 × 10^8^ cells were separated on 12% Tris-glycine gels, immobilized on nitrocellulose membranes and probed with antiserum specific for the antigens indicated on the left. FlaB serves as a loading control to demonstrate equivalent protein amounts between samples. Cell densities are indicated at the top of each lane, and positive controls for the A3*ntrA* samples are indicated by a plus sign (+).

Cultures were inoculated in 1 l of fresh Barbour–Stoenner–Kelly (BSKII) medium at 4 × 10^6^ cells ml^−1^, and aliquots were harvested as the spirochete density increased. The RNA was purified and the levels of *rpoS* and *ospC* transcripts were measured, normalized to *flaB* as an internal control and compared with the levels in the initial inoculums. Interestingly, *rpoS* transcript levels increased 2- to 4-fold in all three *B. burgdorferi* strains in samples harvested at 1 × 10^7^, 2 × 10^7^ and 5 × 10^7^ cells ml^−1^ in comparison with the inoculums (Fig. 6A). Levels of *rpoS* transcript decreased in all three strains as cell density increased to 1–2 × 10^8^ cells ml^−1^, and then increased markedly in the B31-A3 and A3*hk2*, but not in A3*ntrA*, as the cultures reached higher densities (2 × 10^8^ cells ml^−1^ + 24 h). Consistent with these results, the *ospC* transcript levels followed similar trends in all three strains (Fig. 6B). Analysis of the *rpoS* and *ospC* transcript levels in A3*ntrA* compared with B31-A3 as cell density increased revealed roughly a 2- to 15-fold decrease in *rpoS* and a 10- to 200-fold decrease in *ospC* transcripts (Fig. 6C). These results are consistent with previous findings demonstrating decreased *rpoS* transcript and protein levels in strains lacking σ^54^ (Hübner *et al*., 2001; Fisher *et al*., 2005; Smith *et al*., 2007). In contrast, comparison of *rpoS* and *ospC* transcripts in A3*hk2* to B31-A3 demonstrated elevated *rpoS* (2- to 5-fold) and *ospC* (4- to 44-fold) transcript levels in A3*hk2* at increased cell densities with the exception that at 2 × 10^8^ cells ml^−1^ + 24 h, the *rpoS* transcript level was approximately the same in both strains (Fig. 6D).

In order to determine protein levels, immunoblot analyses were used to examine RpoS and OspC expression in the three *B. burgdorferi* strains as cultures transitioned from lower to higher cell density. In comparison with the initial inoculums, the RpoS levels in B31-A3 and A3*hk2* initially increased, then decreased, and then increased again as cultures reached maximum cell density (Fig. 6E). In contrast, while RpoS was shown to be virtually undetectable in A3*ntrA* at low and high cell densities, trace amounts appeared to be detectable at moderate cell density (2 × 10^7^−1 × 10^8^ cells ml^−1^). OspC was undetectable at all cell densities in A3*ntrA* (Fig. 6E). Taken together, these results indicate that although *rpoN* is required for *rpoS* expression as cell density increases, it appears that *hk2* is not essential.

### Influence of *hk2* and *rpoN* mutations on *rpoS* and *ospC* mRNA and protein levels in *B. burgdorferi* cultures following an increase in growth temperature

**Fig. 7:**
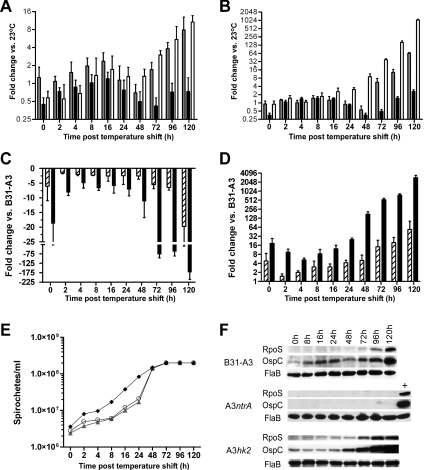
Quantitative RT-PCR analysis of *rpoS* and *ospC* transcripts and immunoblot analysis of RpoS and OspC following an increase in growth temperature from 23°C to 34°C. RNA was extracted from *B. burgdorferi* strains B31-A3 (grey bars), A3*ntrA* (black bars) and A3*hk2* (white bars) grown at 23°C and following a temperature shift to 34°C, and transcripts were quantified using specific primers and probes with the Taqman system. Values have been normalized to the internal control, *flaB.* Data presented represents averages of three assays performed in quadruplicate. Error bars represent standard deviation. A. QRT-PCR analysis of *rpoS* following a temperature shift. Fold changes are expressed relative to spirochetes grown at 23°C. B. QRT-PCR analysis of *ospC* following a temperature shift. Fold changes are expressed relative to spirochetes grown at 23°C. C. QRT-PCR analysis of *rpoS* (hatched bars) and *ospC* (black bars) transcripts in A3*ntrA* relative to B31-A3. Fold changes are expressed compared with the B31-A3 at corresponding time points. D. QRT-PCR analysis of *rpoS* (hatched bars) and *ospC* (black bars) transcripts in A3*hk2* relative to B31-A3. Fold changes are expressed compared with the B31-A3 at corresponding time points. E. Growth curves of B31-A3 (grey triangles), A3*ntrA* (black diamonds) and A3*hk2* (open circles) following a temperature shift from 23°C to 34°C. F. Immunoblot analysis of RpoS and OspC levels in *B. burgdorferi* strains B31-A3, A3*ntrA* and A3*hk2* following an increase in growth temperature from 23°C to 34°C. Whole-cell lysates of *B. burgdorferi* strains equivalent to approximately 8 × 10^7^−1 × 10^8^ cells were separated on 12% Tris-glycine gels, immobilized on nitrocellulose membranes and probed with antiserum specific for the antigens indicated on the left. FlaB serves as a loading control to demonstrate equivalent protein amounts between samples. Time points are indicated at the top of each lane, and positive controls for the A3*ntrA* samples are indicated by a plus sign (+).

A previous study has shown that *B. burgdorferi* strain 297 *rpoS* transcript levels increase 2-fold in response to a temperature shift from 23°C to 37°C, 6-fold upon entry into stationary phase, and as much as 40- to 70-fold following several days growth at 37°C (Caimano *et al*., 2004). In order to examine the role of an *hk2* mutation on the transcription of *rpoS* and *ospC* following a shift in growth temperature from 23°C to 34°C, both *rpoS* and *ospC* transcript levels were monitored using QRT-PCR in *B. burgdorferi* B31-A3, A3*ntrA* and A3*hk2*. At various time points following temperature elevation, spirochetes were harvested, RNA was purified, and the levels of *rpoS* and *ospC* transcripts were subsequently determined and compared with transcript levels from cultures grown at 23°C. As shown in Fig. 7A, when compared with cultures maintained at 23°C, both B31-A3 and A3*hk2* showed increased *rpoS* transcript levels (approximately 2-fold) at 16 h post temperature shift. By 120 h post temperature shift, *rpoS* transcript levels increased 7- to 10-fold in B31-A3 and A3*hk2*. Similarly, *ospC* transcript levels increased approximately 8-fold in B31-A3 and A3*hk2* by 72 and 48 h post temperature shift respectively, and continued to increase at the time points of 96 and 120 h (Fig. 7B). Interestingly, the observed increase in *ospC* transcript in A3*hk2* (1000-fold) was much greater than that observed in B31-A3 (64-fold) at 120 h post temperature shift. As expected, both the *rpoS* and *ospC* transcripts in A3*ntrA* remained at similar levels to a culture at 23°C for the duration of the experiment (Fig. 7A and B). Analysis of the *rpoS* and *ospC* transcript levels in A3*ntrA* compared with B31-A3 following a temperature shift revealed a 2- to 20-fold decrease in *rpoS* and a 5- to 200-fold decrease in *ospC* transcripts (Fig. 7C). In contrast, comparison of *rpoS* and *ospC* transcripts in A3*hk2* versus B31-A3 demonstrated markedly increased *rpoS* (2- to 60-fold) and *ospC* (4- to 2000-fold) transcript levels in A3*hk2* (Fig. 7D). Growth curves relating cell densities with time points post temperature shift are shown (Fig. 7E).

Immunoblot analyses were employed to examine RpoS and OspC expression in the three *B. burgdorferi* strains following an increase in growth temperature. Both RpoS and OspC levels in B31-A3 were initially almost undetectable but increased with time as cultures were incubated at 34°C, and both proteins were clearly evident by the 120 h post-temperature shift (Fig. 7F). A3*hk2* demonstrated a similar pattern of RpoS and OspC expression to B31-A3 in that both proteins increased following the temperature shift; however, the levels of protein expressed by A3*hk2* appeared to be slightly higher. As expected, RpoS and OspC were virtually undetectable in A3*ntrA* at all time points (Fig. 7F). Overall, these QRT-PCR and immunoblotting data support previous studies indicating that *rpoN* is required for *rpoS* expression following a temperature shift from 23°C to 34°C, and demonstrate that *hk2* does not appear to be required for this adaptive response to occur.

### Rrp2 requires *rpoN*, but not *hk2* for transcriptional activation from the *rpoS* promoter in *B. burgdorferi*

**Fig. 8:**
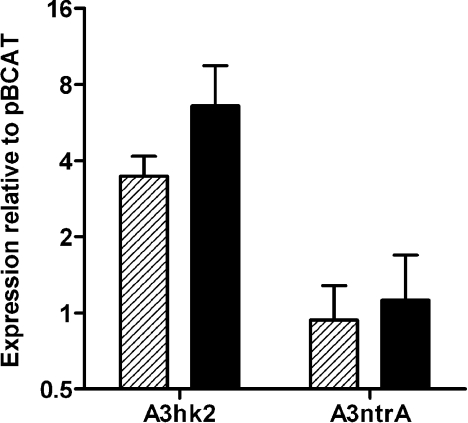
Transcript levels of *cat* in *B. burgdorferi* A3*ntrA* and A3*hk2* as measured by QRT-PCR. *cat* transcripts levels were measured in *B. burgdorferi* A3*hk2* and A3*ntrA* harbouring plasmids pMB313 (hatched bars) and pMB92S (black bars). Fold changes are relative to strains harbouring pBCAT. All values have been normalized to the internal control, *flaB*. Data presented represents averages of three assays performed in quadruplicate. Error bars represent standard deviation.

To investigate whether the rpoSP-*cat* expression was dependent on σ^54^ and Hk2, both pMB313 and pMB92S reporter plasmids were transformed into *B. burgdorferi A3ntrA* and A3*hk2*. QRT-PCR was employed to determine the levels of *cat* transcript when cells reached high density (2 × 10^8^ cells ml^−1^) from strains containing pMB313 and pMB92S compared with strains harbouring vector alone. Results showed that *cat* transcript levels in A3*hk2* increased approximately 4- to 8-fold compared with the vector only control (Fig. 8). In contrast, no appreciable increase in *cat* transcript levels was evident in A3*ntrA* consistent with decreased *rpoS* transcript levels in this mutant. The results obtained for A3*hk2* are similar to the results observed for wild-type B31-A3 (Fig. 4A), and suggest that *hk2* is not absolutely required for transcriptional activation from the σ^54^-dependent *rpoS* promoter.

## Discussion

Signal transduction pathways are important mechanisms for bacteria to sense, respond and adapt to changing environmental stimuli. In the complex life cycle of *B. burgdorferi*, the σ^54^–σ^S^ regulatory cascade plays a critical role in the differential expression of a variety of lipoproteins associated with host adaptation and virulence (Elias *et al*., 2000; Hübner *et al*., 2001; Yang *et al*., 2003a,b; 2005; Fisher *et al*., 2005). It has been previously demonstrated that the two-component response regulator Rrp2 serves as the activator for this regulatory pathway (Yang *et al*., 2003a). In the present study, we confirmed transcription of *rpoS* from its σ^54^-dependent promoter and showed that Rrp2 activates *rpoS* transcription from this promoter.

Despite the fact that the σ^54^-dependent promoter of *B. burgdorferi rpoS* is a near-perfect match to the consensus sequence and would, therefore, be expected to have a high affinity for σ^54^-RNAP holoenzyme, QRT-PCR assays indicate that *rpoS* transcript levels are relatively low in *B. burgdorferi*, ranging from ∼1% to 4% of those for *flaB*. Consistent with previous reports, our results show that *rpoS* mRNA levels increase only 4- to 5-fold in *B. burgdorferi* as cultures transition from lower to higher cell density when grown at 34°C, and only about 2-fold immediately following a temperature shift from 23°C to 34°C. Moreover, even 5 days (120 h) post temperature shift when spirochetes had reached maximal cell density, we observed only a 7- to 10-fold increase in *rpoS* transcript levels. We postulate that the modest level of transcription initiating from the σ^54^-dependent *rpoS* promoter was because Rrp2 does not bind a nearby enhancer to activate transcription from this promoter. Two important findings from this study support this hypothesis. First, we observed that neither purified Rrp2 nor Rrp2Δ123 bound to DNA fragments up to 238 bp upstream and 59 bp downstream of the *rpoS* promoter in mobility-shift DNA binding assays using a variety of conditions. Second, *in vivo* investigations employing promoter–reporter gene fusion assays in both *E. coli* and *B. burgdorferi* revealed that neither DNA sequences upstream nor downstream of the *rpoS* promoter were required for transcriptional activation by Rrp2.

The predicted domain structure of Rrp2 indicates that it contains an amino-terminal receiver domain, a central ATPase domain and a carboxy-terminal domain with a potential helix–turn–helix DNA binding motif (Yang *et al*., 2003a). Most σ^54^-dependent activators contain a carboxy-terminal DNA binding domain that is responsible for enhancer recognition; examples include NtrC, NtC1 and DctD (Reitzer and Magasanik, 1986; Lee and Hoover, 1995; Doucleff *et al*., 2005). However, there are σ^54^-dependent activator proteins that lack a DNA binding domain, including *Helicobacter pylori* FlgR and *Chlamydia trachomatis* CtcC (Koo and Stephens, 2003; Brahmachary *et al*., 2004). In the case of FlgR, it has been shown that this activator does not require an upstream enhancer, but rather appears to contact σ^54^-holoenzyme directly to activate transcription (Brahmachary *et al*., 2004). Although Rrp2 does not appear to bind to a specific enhancer near the *rpoS* promoter to activate transcription, it may bind to low-affinity sites or contact the closed complex at the *rpoS* promoter directly from solution to activate transcription. Rrp2 did bind to heparin–sepharose during purification procedures, suggesting that its DNA binding domain is functional and further supporting the idea that Rrp2 displays non-sequence specific DNA binding.

Efforts to isolate *B. burgdorferi rrp2* mutants have been unsuccessful. Yang and colleagues (2003a) have also reported on the inability to inactivate *rrp2*, but were able to replace the wild-type *rrp2* allele with a mutant allele that presumably prevented Rrp2 from activating transcription while not have interfering with other activities of the protein such as DNA binding. Our inability to inactivate *rrp2* does not appear to be due to polar effects on downstream genes because *rrp2* is predicted to be the last gene within the putative *rpoS* operon. We postulate that Rrp2 has an additional, essential role in the cell that is unrelated to its function in activating transcription from *rpoS* or any other σ^54^-dependent gene. One vital function that Rrp2 may play in the cell is to repress one or more genes whose unregulated expression would otherwise compromise the viability of the cell. Studies are ongoing to identify specific Rrp2 binding sites within the *B. burgdorferi* genome that might help shed light on a potential repressor role for Rrp2.

Analysis of *rpoS* and *cat* transcript levels in *B. burgdorferi rpoN* and *hk2* mutant strains revealed that σ^54^ is required for *rpoS* expression as previously reported, but that *hk2* does not appear to be involved in cell density- or temperature-dependent regulation. When compared with both wild-type and the *rpoN* mutant, expression of *rpoS* mRNA was higher in the *hk2* mutant, a similar trend was observed with *cat* transcript levels. We propose that Hk2 is dispensable for expression of *rpoS* because Rrp2 may be able to accept phosphate from another protein histidine kinase or a low-molecular-weight phosphate donor, such as acetyl phosphate, in the cell as reported for other systems (Lukat *et al*., 1992; Da Re *et al*., 1999; Yamamoto *et al*., 2005). It is unclear exactly what signal(s) lead to activation of Rrp2 or what cellular factors interact with Rrp2, but the ability of Rrp2 to accept phosphate from alternative donors could provide multiple sources of signal input and greater flexibility in regulating the σ^54^ and σ^S^ regulons in *B. burgdorferi.* Interestingly, *rpoS* transcript levels in the *hk2* mutant were slightly higher than wild-type levels. This may be due to altered expression of *rrp2* in the *hk2* mutant. Alternatively, some histidine kinases stimulate the removal of phosphate from their cognate phosphorylated response regulators (Keener and Kustu, 1988). If Hk2 has such activity, then Rrp2-phosphate may accumulate to higher levels in the *hk2* mutant, which could account for increased *rpoS* transcript levels in the mutant.

**Fig. 9:**
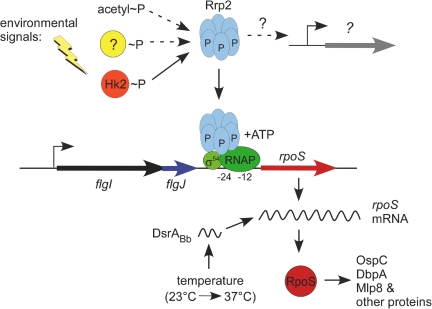
Proposed model for the regulation of *rpoS* in *B. burgdorferi*. Environmental signals are sensed by Hk2 (orange circle) leading to autophosphorylation and subsequent phosphotransfer to the receiver domain of Rrp2 (blue ovals). Alternately, other sensor kinases (yellow circle with question mark)(e.g. BB0420 or BB0363) or small phosphate donors, such as acetyl-phosphate (acetyl∼P), could activate Rrp2, which, in turn, would activate the transcription of *rpoS* (red arrow) from the σ^54^-dependent promoter (indicated by −24/−12). RNAP is represented as a green oval, σ^54^ is shown as small green circle, and RpoS is represented as a red circle. The genes upstream of *rpoS* are shown as follows: *flgI* (black arrow) and *flgJ* (blue arrow). It is possible that Rrp2 could interact with other genes of unknown function (grey arrow with question mark above). Putative promoters are indicated as small bent black arrows. A small RNA molecule (DsrA_Bb_) is shown as a wavy line and is proposed to regulate the efficiency of *rpoS* mRNA (long wavy line) translation in response to temperature (Lybecker and Samuels, 2007). The described model suggests that multiple signals could affect the regulation of the σ^S^ regulon.

Based upon our present results in combination with previously published studies, we propose a model for the regulation of *rpoS* in *B. burgdorferi* (Fig. 9). In brief, signals are sensed by Hk2 or other histidine kinases (e.g. BB0420, annotated as a sensory transduction protein kinase; BB0363, annotated as a sensory box/GGDEF family protein, ERGO genome database, Integrated Genomics), triggering autophosphorylation and subsequent transfer of a phosphate to the N-terminal receiver domain of Rrp2. Alternately, Rrp2 may accept a phosphate from a low-molecular-weight phospho-donor(s) (e.g. acetyl phosphate). After activation, Rrp2 interacts with σ^54^-RNAP holoenzyme bound to the −12/−24 promoter region upstream of *rpoS* and activates transcription leading to expression of σ^S^-controlled genes (e.g. *ospC*, *dpbA*, *mlp8*). If correct, this model would permit multiple signals to be channelled through Rrp2 to activate the σ^54^–σ^S^ regulatory network.

## Experimental procedures

### Strains, plasmids and culture conditions

The strains and plasmids used in this study are shown in Table 1. *E. coli* strains were grown in Luria–Bertani (LB) broth or on LB agar at 37°C. *B. burgdorferi* strain B31-A3 and derivatives were grown in modified BSKII medium pH 7.6 (Barbour *et al*., 1984) or on BSKII agar plates at 34°C unless otherwise stated. Spirochete numbers were determined by dark field microscopy. When necessary, antibiotics were added to the media at the following concentrations: for *E. coli*, ampicillin, 100 μg ml^−1^; chloramphenicol, 25 μg ml^−1^; kanamycin, 25 μg ml^−1^; gentamicin, 10 μg ml^−1^; and for *B. burgdorferi*, rifampicin, 10 μg ml^−1^; amphotericin B, 7.5 μg ml^−1^; kanamycin, 200 μg ml^−1^; gentamicin, 40 μg ml^−1^. All chemicals were purchased from Sigma (St Louis, MO) unless otherwise stated.

**Table 1:** Strains and plasmids used in this study.

Strain/Plasmid	Description/relevant features^a^	Reference
Strain		
*E. coli* strains		
Top 10	General cloning strain	Invitrogen
BW25113	Red recombinase mutagenesis strain	Datsenko and Wanner (2000)
ΔrpoN-Cm	BW25113 *rpoN* deletion strain; Cm^R^	This study
*B. burgdorferi* strains		
B31-A	Clone A, high-passage, avirulent	Rosa *et al*. (1996)
B31-ARpoN	*rpoN* mutant of B31-A, Km^R^	This study
B31-A3	Wild-type clone A3, low-passage, virulent	Elias *et al*. (2002)
A3*ntrA*	*rpoN* mutant of B31-A3, Km^R^	Fisher *et al*. (2005)
A3*hk2*	*hk2* mutant of B31-A3, Km^R^	This study
Plasmids		
*E. coli* plasmids		
pLysE	Constitutively expresses T7 lysozyme; Cm^R^	Invitrogen
pPBMB101	Promoter-less *lacZ* reporter vector, p15A ori; Km^R^	P.J. Brett (unpublished)
pBAD-TOPO	Arabinose-inducible expression vector, pBR322 ori; Ap^R^	Invitrogen
prrp2op	pUC57::*rrp2op*; Ap^R^	GenScript Corporation
pBADrrp2op	pBAD-TOPO::*rrp2op*; Ap^R^	This study
pBADrrp2opΔ123	pBAD-TOPO::*rrp2op*Δ123; Ap^R^	This study
prpoSP_313_	pPBMB101::rpoSP_313_; Km^R^	This study
prpoSP_141_	pPBMB101::rpoSP_141_; Km^R^	This study
prpoSP_121_	pPBMB101::rpoSP_121_; Km^R^	This study
prpoSP_92_	pPBMB101::rpoSP_92_; Km^R^	This study
prpoSP_92S_	pPBMB101::rpoSP_92S_; Km^R^	This study
prpoSP_92S2_	pPBMB101::rpoSP_92S2_; Km^R^	This study
prpoSP_92SGG_	pPBMB101::rpoSP_92SGG_; Km^R^	This study
pbosRPO	pPBMB101::bosRPO; Km^R^	This study
pJLB12a	P_flaB_-kan cassette	Bono *et al*. (2000)
pKD3	Cm^R^ cassette for construction of ΔrpoN-Cm	Datsenko and Wanner (2000)
pPCRscriptCamSK +	Cloning vector, pBC SK(+) derivative, pUC ori, Cm^R^	Stratagene
*B. burgdorferi* plasmids		
pBSV2G	Borrelia shuttle vector; Gm^R^	Elias *et al*. (2003)
pBCAT	pBSV2G::′*cat*; Gm^R^	This study
pMB313	pBCAT::rpoSP_313_; Gm^R^	This study
pMB92S	pBCAT::rpoSP_92S_; Gm^R^	This study
pHk2	pPCRScriptCamSK + ::BB0764 ORF; Cm^R^	This study
pSKHk2	pHk2::P_flaB_-kan; Cm^R^ Km^R^	This study

a Antibiotic resistance: Ap, ampicillin; Cm, chloramphenicol; Gm, gentamicin; Km, kanamycin.

For *rpoS* and *ospC* transcript and protein-level quantification experiments, *B. burgdorferi* starter cultures were grown at 34°C from glycerol stocks. These cultures were diluted to ∼10^5^ cells ml^−1^ in 200 ml of BSKII and incubated at 34°C until a density of ∼2–4 × 10^7^ cells ml^−1^ was reached. Cultures were used to inoculate 1 l of BSKII at a density of ∼4 × 10^6^ cells ml^−1^ and incubation continued at 34°C. At 8–12 h intervals, spirochetes were enumerated and aliquots were harvested for RNA isolation or protein analysis.

Temperature-shift experiments were performed based on previously described methods (Ojaimi *et al*., 2003; Caimano *et al*., 2004). Briefly, *B. burgdorferi* strains were inoculated from glycerol stocks, grown to mid-log phase at 34°C, diluted to ∼10^5^ cells ml^−1^ and grown at 23°C to a density of ∼5 × 10^7^ cells ml^−1^. Spirochetes were then diluted to ∼5 × 10^5^ cells ml^−1^ in larger culture volumes and grown at 23°C to a density of 1–3 × 10^7^ cells ml^−1^ (mid-log phase), at which time cells were transferred to pre-warmed BSKII at a density of 2–4 × 10^6^ cells ml^−1^ and further incubated at 34°C. Cells were harvested for RNA isolation at *t* = 0, 2, 4, 8, 16, 24, 48, 72, 96 and 120 h, and for protein analysis at *t* = 0, 8, 16, 24, 48, 72, 96 and 120 h. Cultures were maintained at 23°C and allowed to continue to 7 × 10^7^−1 × 10^8^, at which time cells were harvested for RNA analysis. The doubling times of the spirochetes grown at 23°C and 34°C were 32–36 h and 8–10 h respectively.

### Primer extension analysis

Total RNA was isolated from a *B. burgdorferi* B31-A and B31-ARpoN (2 × 10^8^ cells ml^−1^) using TRI-Reagent (Sigma) as described by the manufacturer. A total of 1 μg of RNA was used as template for primer extension reactions with the Primer Extension System AMV (Promega, Madison, WI). A sequence ladder was generated with the *fmol* Cycle Sequencing System (Promega) using 1 μg chromosomal DNA isolated from *B. burgdorferi* B31-A. For primer extension and sequencing reactions, primers rpoS-4 and up-PE were labelled at the 5′-end with ^32^P[γ]-ATP (3000 Ci mmole^−1^) (ICN, Costa Mesa, CA) using T4 polynucleotide kinase (Promega). Reaction products were separated by electrophoresis on a 6% polyacrylamide, 7 M urea gel. Reaction products were visualized by autoradiography.

### SDS-PAGE and immunoblots

For protein analysis, *B. burgdorferi* whole-cell lysates were prepared by washing spirochete pellets twice with Haley's buffer (20 mM HEPES, pH 7.6, 50 mM NaCl), followed by lysis with 1× Tris-glycine SDS sample buffer (Invitrogen). For immunoblotting, *B. burgdorferi* lysates equivalent to 8 × 10^7^−1 × 10^8^ cells were separated on 12% Tris-glycine polyacrylamide gels (Invitrogen) and electrophoretically transferred to nitrocellulose membranes (0.45 μm pore size, Invitrogen). Immunoblot analyses were performed at room temperature with incubations as follows: membranes were blocked with 5% skim milk in TBS (100 mM Tris-HCl, pH 7.5, 150 mM NaCl) for 30–60 min, followed by application of the primary antibody diluted in TBS-T (TBS, 0.05% Tween 20) for 1 h, then application of the secondary antibody diluted in TBS-T for 1 h. Membranes were washed three times with TBS-T following each incubation step. Blots were visualized with ECL™ Plus Western Blotting Detection Reagents (GE Healthcare) as per the manufacturer's instructions. Primary antibodies included anti-RpoS polyclonal antiserum (1/1200), anti-OspC polyclonal antiserum (1/1000), anti-Rrp2 polyclonal antiserum (1/1000) and anti-FlaB monoclonal antibody MCA9724 (1/1000). Secondary antibodies were either anti-rabbit IgG-HRP conjugate (1/5000) or anti-mouse IgG-HRP conjugate (1/5000). When appropriate, nitrocellulose membranes were stripped with Restore™ Western Blot Stripping Buffer (Pierce) and washed with TBS-T prior to reprobing.

*Escherichia coli* lysates and purified Rrp2 proteins were separated on 4–20% Tris-glycine polyacrylamide gels (ISC Bioexpress, Kaysville, UT) and stained with Coomassie brilliant blue or analysed by Western blot as described above.

### Cloning and expression of *rrp2op* and *rrp2opΔ123*

For expression in *E. coli*, a codon-optimized version of *B. burgdorferi rrp2* (designated *rrp2op*) was synthesized and cloned into pUC57 by GenScript Corporation (Piscataway, NJ). Full-length *rrp2op* (1356 bp) was PCR amplified from plasmid prrp2op using the Rrp2op-F and Rrp2op-R primer pair. A truncated version of *rrp2* (987 bp) lacking the N-terminal 369 bp, designated *rrp2opΔ123*, was similarly PCR amplified using the Rrp2opΔ123-F and Rrp2op-R primer pair. The PCR products were individually cloned into the pBAD-TOPO vector (Invitrogen) to place the *rrp2* alleles under control of the *araBAD* promoter/operator. DNA sequencing was used to confirm that the *rrp2* gene sequences in the resulting plasmids, pBAD*rrp2op* and pBAD*rrp2opΔ123*, were correct.

### Purification of Rrp2 and Rrp2Δ123

Rrp2 and Rrp2Δ123 were overexpressed in *E. coli* Top 10 (pLysE) cells. Bacteria were grown in 500 ml of LB medium with appropriate antibiotics at 37°C with aeration. When cells reached an OD_600_ of 0.6, expression of protein was induced using 0.02% l-arabinose for 18 h. Cells were harvested by centrifugation, resuspended in buffer A (20 mM Tris pH 8, 50 mM KCl, 5% glycerol, 0.5 mM DTT) and lysed by sonication. Insoluble material was removed by centrifugation, and the supernatant was applied to a Heparin Sepharose™6 Fast Flow (GE Healthcare, Uppsala, Sweden) column. The protein was eluted with a stepwise gradient of KCl (200 mM, 300 mM, 400 mM, 500 mM, 750 mM and 1 M KCl). Fractions were analysed by SDS-PAGE, and those containing Rrp2 or Rrp2Δ123 were pooled, concentrated and stored in buffer A at 4°C. Protein concentrations were determined using a BCA™ protein assay kit (Pierce Biotechnology, Rockford, IL). Purified protein was used in mobility-shift DNA binding assays.

### Polymerase chain reaction, RT-PCR, QRT-PCR and DNA mobility-shift assays

Polymerase chain reactions were performed using the Expand High Fidelity PCR System (Roche Applied Science, Indianapolis, IN) as per the manufacturer's instructions. Genomic DNA used for PCRs was isolated from bacterial strains using the Wizard genomic DNA purification kit (Promega). PCR primers are shown in Table 2. All primers were obtained from Integrated DNA Technologies (Coralville, IA). DNA sequencing was performed by ACGT (Wheeling, IL).

*Borrelia burgdorferi* RNA was extracted using TRI-Reagent (Sigma) as described by the manufacturer. RNA was treated with DNase I and further purified using the RNeasy miniprep kit (Qiagen). cDNA synthesis reactions for RT-PCR and QRT-PCR were performed using SuperScript III (Invitrogen) to synthesize first-strand cDNA following the manufacturer's instructions. RT-PCR primers specific for *rrp2* are shown in Table 2. QRT-PCR primers and probes specific for *cat*, *rpoS*, *ospC* and *flaB* were designed using Primer Express 1.0 and are shown in Table 2. Reactions were performed in a total volume of 20 μl using TaqMan Universal PCR Master Mix (Applied Biosystems, Foster City, CA), 1–2 ng of first-strand cDNA, 300 nM forward and reverse primers, and 250 nM probe. All reactions were carried out on the ABI PRISM 7900HT Sequence Detection System (Applied BioSystems) using a PCR cycle of 2 min at 50°C, 10 min at 95°C, followed by 40 cycles at 95°C for 15 s and 60°C for 1 min. Each transcript was normalized by comparison with the constant, internal control *flaB* (Fisher *et al*., 2005). Three individual assays were performed in quadruplicate.

The *rpoS* promoter/operator (P/O) region was amplified by PCR using the rpoSP313-FB and rpoSP-RXhoI primer pair, generating a 313 bp DNA fragment, or using the rpoSP141-FB and rpoSP-RXhoI primer pair, generating a 141 bp DNA fragment. Mobility-shift DNA binding reactions were performed essentially as previously described (Porter *et al*., 1993; Boylan *et al*., 2003; 2006), or with the Sigma Mobility Shift Optimization Assay kit (Sigma) using purified Rrp2 or Rrp2Δ123 (concentrations ranging from 100 nM to 2 μM) and approximately 20 000 cpm of [γ-^32^P]-ATP-labelled target sequence. Varying concentrations of the components in the binding buffer were also used in attempts to optimize binding conditions. Purified BosR protein and a labelled DNA fragment bearing the *napA*-P/O were employed as a positive control (Boylan *et al*., 2003).

### *Escherichia coli*‘lacZ reporter system

The *B. burgdorferi rpoS*-P/O regions (P_313_, P_141_, P_121_, P_92_, P_92S_ P_92S2_ and P_92SGG_) diagrammed in Fig. 3A were PCR amplified from strain *B. burgdorferi* B31-A3 genomic DNA using one of the following forward primers rpoSP313-FB, rpoSP141-FB, rpoSP121-FB, rpoSP92-FB, rpoSP92S-FB, rpoSP92S2-FB, rpoSP92GG-FB and the reverse primer rpoSP-RXhoI. The *B. burgdorferi rpoS*-P/O regions were cloned upstream of a promoter-less *lacZ* into the BamHI/XhoI sites of pPBMB101 and transformed into the appropriate *E. coli* strain. As a control, the *B. burgdorferi bosR*-P/O region was similarly amplified using the bosRPO-FB and bosRPO-RX primer pair and cloned into the BamHI/XhoI sites of pPBMB101.

An *E. coli rpoN* mutant (ΔrpoN-Cm) was constructed in strain BW25113 via red recombinase mutagenesis (Datsenko and Wanner, 2000) using the EcrpoN-F and EcrpoN-R primer pair. Disruption of *rpoN* was confirmed by PCR using the c1 and yhbG-430 primer pair. The *rpoS-lacZ* reporter plasmids (pPBMB101 derivatives) and arabinose-inducible expression constructs pBAD*rrp2op* or pBAD*rrp2opΔ123* were cotransformed into *E. coli* Top 10 cells or ΔrpoN-Cm cells, and were grown for 6 h at 37°C in the presence or absence of 0.002% l-arabinose. β-Galactosidase (LacZ) assays were performed by the method of Miller (1972) 2–3 times in triplicate.

### Generating a *B. burgdorferi hk2* mutant

To inactivate *hk2*, BB0764 was amplified by PCR from *B. burgdorferi* B31-A3 with primers hk2-BF and hk2-BR. The resulting PCR product was digested with BamHI to produce 1.3 kb fragment containing *hk2*, and was cloned into the BamHI restriction site of pPCRScriptCamSK + (Stratagene, La Jolla, CA), generating the plasmid pHk2. A kanamycin-resistance cassette driven by the *B. burgdorferi flaB* promoter region was amplified from pJLB12a (Bono *et al*., 2000) using primers hk2-SphF and hk2-SphR, which introduced SphI restriction sites on the ends. The PCR product and pHk2 were digested with SphI and ligated together to generate pSKHk2. The resulting plasmid was transformed into low-passage *B. burgdorferi* strain B31-A3, as described by Samuels (1995), and kanamycin-resistant colonies were analysed by PCR using primers hk2-BF and hk2-BR. To assess whether *rrp2* was transcribed in the resulting mutant strain (A3*hk2*), total RNA was isolated, and RT-PCR was performed using the rrp2-RTF and rrp2-RTR primer pair. The plasmid profile of A3*hk2* was assessed as previously described (Purser and Norris, 2000; Elias *et al*., 2002).

### *Borrelia burgdorferi rpoS* promoter–*cat* reporter constructs

A *B. burgdorferi* reporter plasmid was constructed by amplifying a promoter-less chloramphenicol acetyltransferase (*cat*) gene from pPCRScriptCamSK + (Stratagene) and cloning it into the *Borrelia* shuttle vector, pBSV2G. The resulting plasmid was designated pBCAT. The *B. burgdorferi rpoS*-P/O regions (P_313_ and P_92S_) were PCR amplified using either the rpoS313-FH or the rpoS92S-FH primer in combination with the rpoSP92-RXb primer, and then cloned into the HindIII/XbaI sites of the *B. burgdorferi* reporter construct pBCAT. All inserts were confirmed by DNA sequencing. The plasmids pMB313, pMB92S and pBCAT (control) were subsequently transformed into *B. burgdorferi* B31-A3, A3*ntrA* and A3*hk2*. For QRT-PCR experiments, RNA was isolated from strains harbouring *cat* reporter constructs that were grown to appropriate cell densities or at various time points following a temperature shift.
